# Overexpression of CXCL17 increases migration and invasion of A549 lung adenocarcinoma cells

**DOI:** 10.3389/fphar.2024.1306273

**Published:** 2024-02-07

**Authors:** Ekin Koni, Irem Congur, Zeynep Tokcaer Keskin

**Affiliations:** ^1^ Graduate School of Natural and Applied Sciences, Department of Molecular and Translational Biomedicine, Acibadem Mehmet Ali Aydinlar University, Istanbul, Türkiye; ^2^ Faculty of Engineering and Natural Sciences, Department of Molecular Biology and Genetics, Acibadem Mehmet Ali Aydinlar University, Istanbul, Türkiye

**Keywords:** CXCL17, A549, migration, invasion, proliferation

## Abstract

Lung cancer is one of the most frequently diagnosed malignancies and is a widespread disease that affects millions of individuals globally. CXCL17 is a member of the CXC chemokine family that attracts myeloid cells and is associated with the mucosa. CXCL17 can both support and suppress tumor growth in certain types of cancer. A549 LUAD cells were transfected with N-Terminal p3XFLAG-CMV or N-Terminal p3XFLAG-CMV-CXCL17 to establish stably transfected CXCL17-overexpressing cells. Reverse-transcription polymerase chain reaction (RT-PCR) and Enzyme Linked Immunosorbent Assay (ELISA) were performed to verify the levels of CXCL17 mRNA and of CXCL17 protein concentration of stably transfected A549 cells respectively. Wound healing, CCK8, and matrigel invasion assays were performed to assess the effect of CXCL17 overexpression on migration, proliferation, and invasion of A549 cells. When compared to control groups, proliferative capacity of A549 cells were unaffected by CXCL17 overexpression; however, the wound area in the CXCL17 overexpression group had dramatically decreased after 48 h. Similarly, the number of invasion cells was significantly higher in the CXCL17-overexpressing group than in the control ones after 48 h. CXCL17 overexpression significantly increased the ability of A549 cells to migrate and invade, without affecting their proliferative abilities.

## 1 Introduction

Lung cancer is one of the most frequently diagnosed malignancies and is a widespread disease that affects millions of individuals globally, which is still the largest cause of cancer-related death worldwide. This disease occurs when abnormal cells in the lungs grow out of control, frequently creating a tumor that can spread to other body organs (Gridelli et al., 2015). According to a pathological perspective, the two main classifications of lung cancer are small cell lung cancer (SCLC) and non-small cell lung cancer (NSCLC) (Fois et al., 2021). The characteristics of SCLC include an elevated rate of metastasis and proliferation, as well as a favorable early response to radiotherapy and chemotherapy (Wang et al., 2020). NSCLC refers to any form of epithelial lung cancer other than SCLC (PDQ Adult Treatment Editorial Board, 2023). 40% of NSCLC instances are lung adenocarcinomas (LUAD), followed by 25% of squamous cell carcinomas and 12% of large cell carcinomas (Schabath and Cote, 2019). Currently, 85% of lung cancer cases seen globally are caused by NSCLC. Oncologists can now tailor the therapy options due to recent advancements in the understanding of pathways, techniques for identifying genetic lesions that can be treated, and newly developed medications to block the actions of the pathways. The prognosis and available treatments for NSCLC are significantly influenced by the disease stage at the time of diagnosis (Herbst et al., 2018). Moreover, more than 90% of cancer deaths are attributed to metastasis. Metastasis is a systemic condition as opposed to primary tumors, which can frequently be treated with local surgery or radiation. Surgery is the first line of defense against lung adenocarcinoma, followed by chemotherapy and radiation therapy. However, cancer frequently returns despite treatment. In fact, within 5 years of diagnosis, 25% of patients with lung adenocarcinoma will experience the onset of metastatic disease (Wu et al., 2022). CXCL17 is a member of the CXC chemokine family that attracts myeloid cells (Gowhari Shabgah et al., 2022). The mucosal tissues of the lungs, trachea, bronchi, stomach, and intestinal lumens—organs of the respiratory and gastrointestinal tract—structurally express *CXCL17*. As a result, CXCL17 is regarded as a chemokine associated with the mucosa (Choreño-Parra et al., 2020). The angiogenic activity of CXCL17, also known as VCC-1 (VEGF-Coregulated Chemokine 1), encourages tumorigenesis (Li et al., 2021). Immune cells are activated and move toward the site of inflammation when CXCL17 binds to its receptor, starting a signaling cascade (Choreño-Parra et al., 2020). Chemokines have been demonstrated to have a significant impact on how cells enter the tumor microenvironment and control how the body’s immune system responds to all cancer cells (Marcuzzi et al., 2018). Even though numerous studies have demonstrated that CXCL17 is highly expressed in primary tumor samples and cancer cell lines, other studies have demonstrated that this cytokine is underexpressed in cancers. CXCL17 has the ability to both support and suppress tumor growth in certain types of cancer (Gowhari Shabgah et al., 2022). CXCL17 promotes angiogenesis and cell proliferation, which, according to numerous studies, aids in the development of tumors in breast and colon malignancies (Mu et al., 2009). Colorectal, breast, hepatocellular, and type I endometrial cancer all showed significantly lower levels of CXCL17 mRNA or protein compared to type I endometrial cancer and pancreatic intraductal papillary mucinous carcinoma (Weinstein et al., 2006). Additionally, the MDA-MB231 cell line’s tendency for cell migration and proliferation is decreased by the downregulation of the gene (Xiao et al., 2021). When pathogen materials are induced, the expression of CXCL17 at steady levels may increase, which might decrease immune responses to support homeostasis and prevent unfavorable immunological reactions (Liu et al., 2020). Additionally, the MDA-MB231 cell line’s tendency for cell migration and proliferation is decreased by the downregulation of the gene (Xiao et al., 2021). According to studies on CXCL17 gene expression in breast cancer (Hashemi and Khorramdelazad, 2023), a high level of CXCL17 gene expression in patients is associated with a worse overall survival. When pathogen materials are induced, the expression of CXCL17 at steady levels may increase, which might decrease immune responses to support homeostasis and prevent unfavorable immunological reactions (Liu et al., 2020). According to a recent study, CXCL17 may be involved in how lung adenocarcinoma (LUAD) spreads to the spine. The data show that CXCL17 activates Src/FAK signaling and promotes mononuclear macrophage chemotaxis.

The treatment of NSCLC has multiple treatment options according to NCCN guidelines version 1.2024. However, there is still a need for new targeted therapies to prevent the spread and provide a full treatment for lung cancer patients. CXCL17 with all the information provided in the literature has a potential to be a great target for drug candidates.

Online databases UALCAN and GEPIA offer resources for the analysis of gene expression data to researchers studying cancer. According to the UALCAN database, LUAD patients had lower levels of CXCL17 gene expression compared to healthy individuals, whereas the GEPIA database demonstrated that LUAD patients had higher levels of gene expression for CXCL17 relative to normal (Chandrashekar et al., 2017; Tang et al., 2017). The lack of information in the literature and the small sample size can be attributed for the misinterpretation of such information. This can lead to inaccurate or incomplete understanding of the topic. In order to solve this unknown concept, we aimed to investigate how CXCL17 overexpression affects the migration, proliferation and invasion capabilities of A549 LUAD cells *in vitro*. Our results demonstrated that increased levels of CXCL17 significantly enhanced migration and invasion capacities of A549 cells but not proliferation.

## 2. Results

### 2.1 Establishment of CXCL17-overexpressing A549 LUAD cells

**FIGURE 1:**
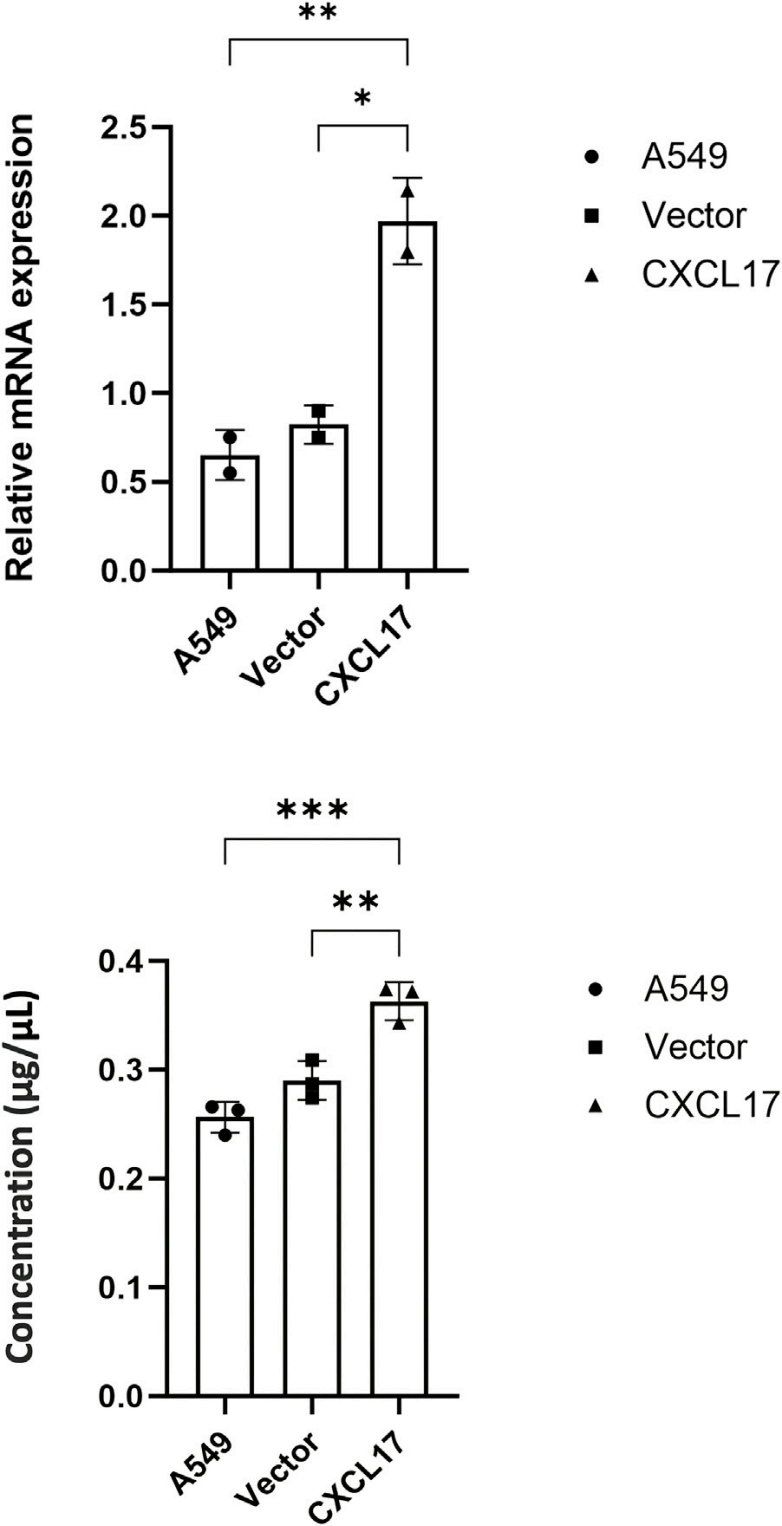
CXCL17 overexpression in CXCL17-gene transfected A549 cells. **(A)** RT-PCR analysis demonstrated the CXCL17 mRNA levels were significantly higher in the CXCL17 group compared to A549 and vector groups. Results are displayed as mean ± SD of two independent experiments. *p*-value<0.05; *0.0145, **0.0098. **(B)** ELISA results showed the protein levels of CXCL17 were significantly higher in CXCL17 group compared to A549 and vector groups. Results are displayed as mean ± SD of two independent experiments. *p*-value<0.05; **0.0039, ***0.0005.

To investigate whether CXCL17 overexpression affects LUAD cell migration, proliferation, and invade, we first transfected A549 cells with N-Terminal p3XFLAG-CMV-CXCL17 or vector only. The mRNA and protein levels of CXCL17 in A549 cells were verified using RT-PCR and ELISA after the development of stable clones with CXCL17 overexpression. The relative mRNA levels in *CXCL17* transfected cells (CXCL17) were significantly higher than untransfected (A549) and vector only transfected (vector) cells having *p*-values of 0.0098 and 0.0145, respectively (Figure 1A). CXCL17 protein concentrations detected by ELISA were significantly higher in the CXCL17 group compared to A549 and vector groups having *p*-values of 0.0005 and 0.0039, respectively (Figure 1B).

### 2.2. CXCL17 overexpression has no effect on the proliferation of A549 cells

**FIGURE 2:**
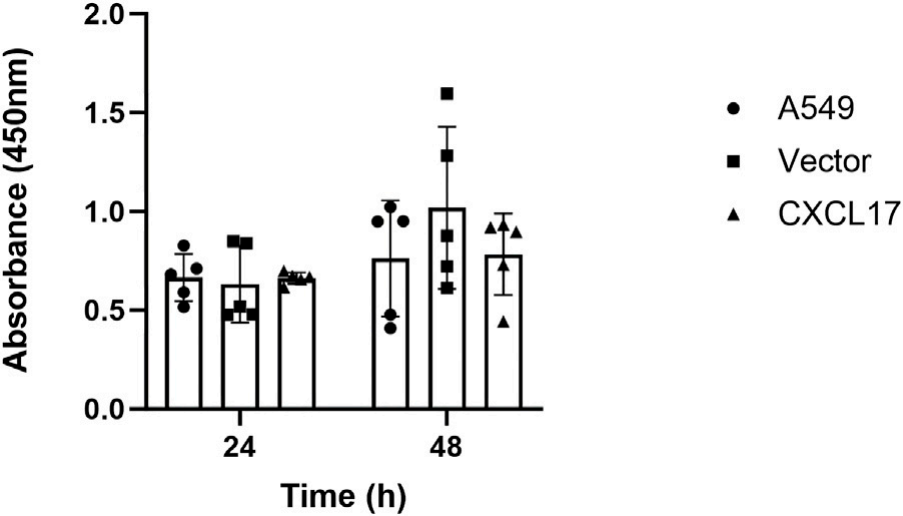
CXCL17 overexpression has no impact on the proliferation of A549 cells. A bar graph displaying each group’s (A549, vector, CXCL17) absorbance at 450 nm after 24 and 48 h. There was no significant difference in the proliferative abilities of these groups at both time points. Results are displayed as mean ± SD of five independent experiments.

We performed CCK8 assay to assess the proliferative capacity in each group in order to examine the effect of CXCL17 overexpression on the proliferation of A549 cells. As shown in Figure 2, A549, vector, and CXCL17 groups all had very similar proliferative capacities at both 24 and 48 h. These findings indicated that overexpression of CXCL17 had no significant effect on the proliferation of A549 cells.

### 2.3 CXCL17 overexpression has an impact on migration abilities of A549 cells

**FIGURE 3:**
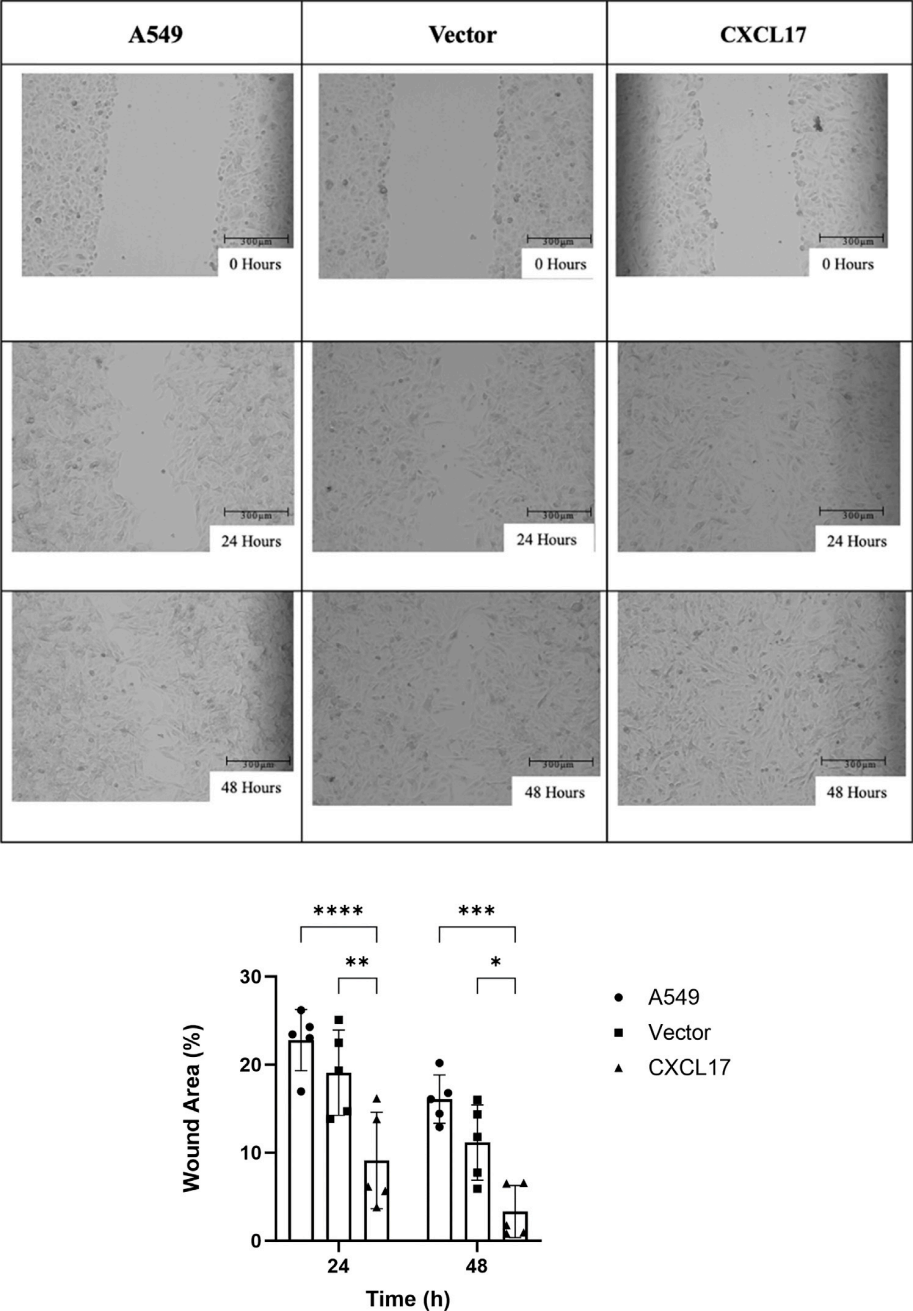
CXCL17 overexpression affects migration of A549 cells. **(A)** The best representation of the wound areas is shown in 10X microscope images taken at three different time intervals (0, 24, and 48 h). Scale bar 300 μm. The ability of cells to migrate had been significantly enhanced in the CXCL17 group. **(B)** A bar graph displaying each group’s (A549, vector, CXCL17) wound area% at 24 and 48 h. Wound area of the CXCL17 group was significantly decreased compared to others. Results are displayed as mean ± SD of five independent experiments. *p*-value<0.05; *0.0153, **0.0021, ***0.0001 ****<0.0001.

In order to determine how CXCL17 overexpression affects the migration of A549 cells, we performed the wound healing assay. As shown in Figure 3A, the CXCL17 group had more than 60% of the scratch’s surface closed after 24 h, compared to 35%–40% for the A549 and vector groups. At the end of 48 h, the wound area of the CXCL17 group significantly decreased as compared to the A549 and vector groups, with *p*-values of 0.0001 and 0.0153, respectively (Figure 3B). These findings suggested that A549 cells had an enhanced capacity for migration when CXCL17 is overexpressed.

### 2.4 CXCL17 overexpression has an impact on invasion capabilities of A549 cells

**FIGURE 4:**
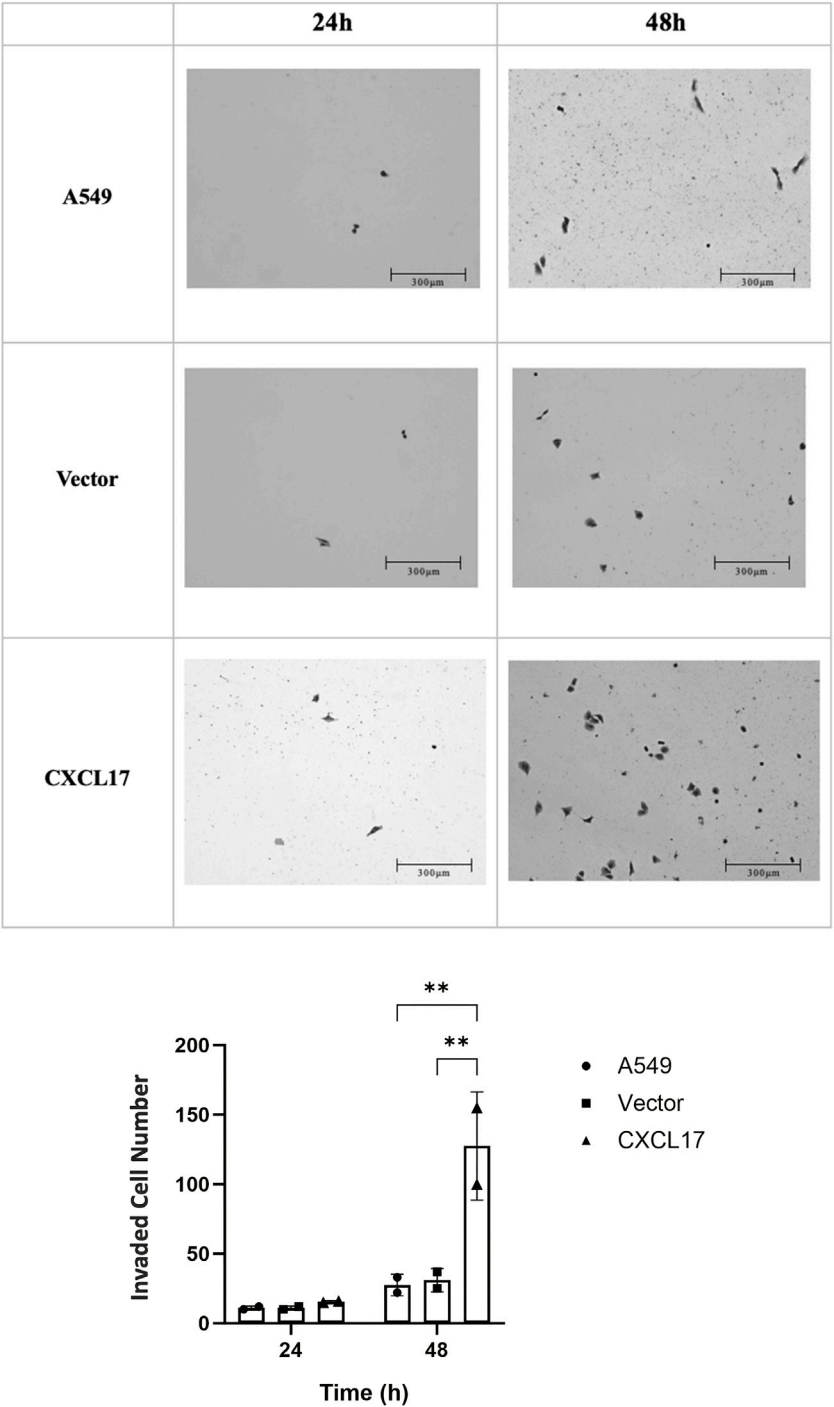
CXCL17 overexpression affects invasion of A549 cells. **(A)** The best representation of the invaded cells is shown in 10X microscope images taken at two different time intervals (24, and 48 h). Scale bar 300 μm. The CXCL17 group demonstrated a markedly increased capacity for cell invasion. **(B)** A bar graph displaying the number of invaded cells in each group (A549, vector, CXCL17). The CXCL17 group had a significantly higher number of invaded cells than the other groups. Results are displayed as mean ± SD of two independent experiments. *p*-value<0.05; **0.0028 and **0.0034, A549 vs. CXCL17 and vector vs. CXCL17, respectively.

To further explore the role of CXCL17, we conducted a Matrigel invasion assay to evaluate the impact of CXCL17 overexpression on the ability of A549 cells to invade. After 24 h, we observed that there was not a significant difference between the number of invaded cells within each group. Nevertheless after 48 h, the CXCL17 group had significantly more invaded cells than the A549 and vector groups, with *p*-values of 0.0028 and 0.0034, respectively (Figure 4). These results demonstrated that overexpressed CXCL17 plays an essential role for cell invasion.

## 3 Discussion

CXCL17 plays a crucial part in angiogenesis, which is essential for supplying nutrients to cancer cells, encouraging cancer growth, and enabling invasion. One of the main methods for managing NSCLC is the current treatment with antiangiogenic drugs such as bevacizumab, which blocks VEGF. Some patients, however, may develop resistance to bevacizumab due to the activation of alternative angiogenic pathways that are unaffected by VEGF inhibition (Russo et al., 2017). The proangiogenic factor FGF signaling, which is controlled by CXCL17, is one of these compensatory mechanisms. Therefore, concentrating on CXCL17 may be a promising strategy to increase the effectiveness of anti-VEGF medications in the treatment of NSCLC (Choreño-Parra et al., 2020). With the UALCAN database analysis (Chandrashekar et al., 2017), we discovered that LUAD patients had significantly lower expression of the CXCL17 gene compared to healthy controls. These findings led us to hypothesize that CXCL17 could promote tumor growth and induce metastasis. As the precise function of CXCL17 in NSCLC is still unclear, in this study, we evaluated the proliferative, migratory, and invasion potential of A549 cells that overexpress CXCL17 to learn more about the effects of CXCL17 in NSCLC cells. Understanding this molecular pathway will facilitate the search for new active compounds that could interact with CXCL17 of some of the proteins that directly or indirectly affects the metastatic properties of lung adenocarcinomas.

Numerous studies show that in addition to promoting the movement of white blood cells, chemokines, like CXCL17, also play a part in a number of physiological processes. In contrast to a new group of macrophage-like cells that were more prevalent, alveolar macrophages were found to be less common in mice lacking the CXCL17 protein. These findings imply that CXCL17 also functions as a novel macrophage chemoattractant in mucosal tissues (Xiao et al., 2021). Breast and colon cancers express CXCL17 at significantly higher levels, where it promotes angiogenesis, the spread of cancerous cells, and the development of the disease (Burkhardt et al., 2014). CXCL17 can promote the growth of SMMC7721 hepatoma carcinoma cells both *in vivo* and in (9). In a recent study, the MTT assay revealed that high levels of *CXCL17* helped the hepatocellular carcinoma cell lines HepG2 and Hepa1-6 survive. This suggests that CXCL17 encourages HCC cell proliferation (Wang et al., 2019). An essential component of tissue formation and regeneration is the control of cell proliferation (Duronio and Xiong, 2013). Therefore, we performed CCK8 assay to evaluate the proliferative capacity of A549 cells. However, in our study it was demonstrated that the proliferative states of the cells were not affected significantly with the overexpression of CXCL17 in A549 cells. In one study, researchers tested the tumorigenic potential of NIH3T3-mouse embryonic fibroblast cells that had CXCL17 overexpressed. They noticed that the level of CXCL17-3T3 cell proliferation was unaffected in comparison to the control under typical adherent culture conditions. However, compared to mock transfected cells, CXCL17-3T3 cells developed tumors more quickly when subcutaneously injected into nude mice (Matsui et al., 2012). This circumstance may also help us explain our findings. We performed wound healing and Matrigel invasion assays to examine the ability of A549 cells to migrate and invade, respectively. Our findings showed that overexpressing CXCL17 significantly increased A549 cells’ migration and invasion. In addition to contributing to the immune response, CXCL17 affects cellular migration which is the onset of diseases such as inflammation and tumor metastasis. Cell migration is, in fact, a crucial component of many physiological phenomena. Invasion, migration, and cell viability play important roles in the pathogenesis of cancer and other biological processes. Cancer metastases are formed when cancer cells disseminate to distant organs as a result of numerous stochastic and complex events. These cells may disperse to distant organs on their own or in response to pressure from the outside world. The ability of the cells to move is a crucial component of cancer metastases (Fares et al., 2020). Moreover, as our results regarding proliferation clearly indicated that CXCL17 overexpression did not change proliferation, wound healing and transwell assay results could be purely attributed to the intrinsic mechanisms that change the mobility of the cells. Wang et al., 2019, demonstrated in hepatocellular carcinoma that CXCL17 promoted this activity via autophagy.Many tumors have been shown to overexpress CXCL17 as a result of proangiogenic agents such as VEGF, CXCL1, and CXCL8. TAMs are immune cells that are activated by high CXCL17 levels in lung adenocarcinomas through the Src/FAK pathway. Then, these TAMs promote the growth and metastasis of malignancies by cultivating an environment that suppresses immunity. Thus, focusing on CXCL17 or its downstream Src/FAK pathway components could be one strategy for treating lung cancer (Hashemi and Khorramdelazad, 2023).

Our research shows that CXCL17 stimulates lung cancer tumor growth and spread. These results imply that CXCL17 might be a viable target for novel cancer treatments. Our findings are in line with earlier studies that demonstrate CXCL17 has a function in tumor development and progression and is overexpressed in a number of cancer types. However, our research is the first to demonstrate that the overexpression of CXCL17 encourages the A549 lung cancer cells to migrate and invade.

A recent study performed in colorectal cancer (CRC) cells used highly expressed GPR35 and CXCL17 in drug-resistant tumor cells. GPR35 expression was shown to be significantly reduced upon CXCL17 knockdown (Bu et al., 2023). In oral squamus cell carcinoma one recent study showed the inhibition of CXCL17 and MUC1 by *Porphyromonas gingivalis* (Lan et al., 2023). Another study performed with HPV-associated cervical cancer pinpointed CXCL17 in relation with Akt pathway and tumor progression (Olwal et al., 2023). The high expression of CXCL17 was also linked with glioblastoma prognosis (Wang et al., 2023). With all the research related with different cancer types show the involvement and the importance of CXCL17 in tumor progression which makes CXCL17 a possible target of candidate drugs and the overexpression model we generated could be a good *in vitro* system to analyze the active compounds and drug candidates while investigating the properties of the drugs and their action mechanisms.

Our studies were conducted *in vitro*, that’s why it is possible that the findings may not fully recapitulate the molecular interactions *in vivo* settings. Moreover, in this study the subcellular localization of CXCL17 and its direct effect in wound healing and invasion was not shown due to study design. Despite these limitations, the results of this study provide valuable insights into the role of CXCL17 gene in NSCLC. Further research, including *in vivo* experiments and studies to reveal the molecular mechanisms, are required to fully understand the molecular interactions and the role of this gene in NSCLC.

To conclude overexpressing CXCL17 in A549 cells did not increase their capacity for proliferating *in vitro*, however, it did increase their capacity to migrate and invade.

## 4 Materials and methods

### 4.1 Cell culture

The human non-small cell lung cancer cell line A549 was cultured in high glucose DMEM (Gibco™, catalog no. 11965092) supplemented with 10% fetal bovine serum (FBS) (Gibco™, catalog no. 10270106) and 1% pen-strep (Gibco™, catalog no. 15140122) and incubated at 37°C, 5% CO_2_.

### 4.2 Reverse transcription polymerase chain reaction

From the ENSEMBL database (https://www.ensembl.org/index.html) (Cunningham et al., 2021), *CXCL17* cDNA sequences were acquired. To be able to select a restriction enzyme to avoid digestion of cDNA sequence, restriction enzymes having “0 cutter” were examined in the NEBcutter software (http://nc2.neb.com/NEBcutter2/) (Vincze et al., 2003). The N-Terminal p3XFLAG-CMV plasmid-compatible restriction enzymes *Eco*RI and *Kpn*I have been chosen as appropriate enzymes for the CXCL17 gene.

Total RNA from A549 cell line was isolated using the iNtRON Biotechnology RNA-spinTM Total RNA Extraction Kit (for Cell/Tissue) (Catalog no. 17211). The cDNA of A549 RNA was generated from 1 µg total RNA per sample using Vazyme HiScript III RT SuperMix for qPCR (+gDNA wiper) kit (Catalog no. R323). New England BioLabs Taq DNA Polymerase with Standard Taq (Mg-free) Buffer kit (Catalog no. M0320S) was used to amplify the CXCL17 gene from 1 µg cDNA using QIAmplifier 96 PCR equipment. *Cyclophilin A* was used as a positive control.

The sequence of primers (Thorvacs Biotechnology LLC) used:

*CXCL17* Forward: 5′GCG​AAT​TCA​AAA​GTT​CTA​ATC​TCT​TCC​CTC​CTC​CT 3′

*CXCL17* Reverse: 5′GCG​GTA​CCC​TAC​AAA​GGC​AGA​GCA​AAG​CTT​C 3′

*CYCLOPHILIN A* Forward: 5′AAT​GGC​ACT​GGT​GGC​AAG​TC 3′

*CYCLOPHILIN A* Reverse: 5′ GCT​CCA​TGG​CCT​CCA​CAA​TA 3′

FLAG TAG Forward: 5′ GAC​TAC​AAA​GAC​CAT​GAC​GGT 3′

### 4.3 Establishment of CXCL17-overexpressing cells

N-Terminal p3xFLAG-CMV vector was used to establish stably transfected *CXCL17*-overexpressing cells. To construct the N-Terminal p3XFLAG-CMV plasmid, the consensus coding sequence (CCDS) of human CXCL17 gene was amplified with RT-PCR from RNA obtained from A549 cells with primers omitting ATG start codon to enable FLAG fusion. The product of this amplification was digested with *Eco*RI and *Kpn*I and inserted into an *Eco*RI- and *Kpn*I -cleaved N-Terminal p3XFLAG-CMV vector and ligated with T4 DNA Ligase according to the manufacturer’s protocol. Restriction enzymes and T4 DNA ligase were purchased from New England Biolabs. The engineered plasmids were sent to Sanger sequencing to verify the correct clone.

jetOPTIMUS^®^ transfection reagent (Catalog no. 101000051) was used for the transfection of cells. A549 cells were transfected with N-Terminal p3XFLAG-CMV or N-Terminal p3XFLAG-CMV-CXCL17 using 10 μg DNA and 10 μL JetOPTIMUS reagent, according to the manufacturer’s protocol (Catalog no. 101000051). 3 days post transfection, cells were treated with 700 μg/mL G418 to select stable transfected cells. Medium was changed every 3 days until all the cells that were not transfected died.

After selection, all experiments were performed on 3 groups: The CXCL17 overexpressing cells (will be referred as CXCL17) The cells transfected with unmodified vector only (will be referred as vector) and untransfected naive A549 cells (will be referred as A549).

### 4.4 Enzyme linked immunosorbent assay

In order to extract protein from A549 cells, 200 μL of RIPA buffer (ABT, catalog no. B08-01-01) containing 1X protease inhibitor cocktail (BOSTER, catalog no. AR1182) was used. The BCA assay (Thermo Fisher Scientific, catalog no. 23225) was used to calculate the total protein concentration. For the purpose of confirming overexpression from cell lysates, the ELK Biotechnologies ELISA Kit (Catalog no. ELK3130) was used. The experiment was conducted in accordance with the manual for the kit’s instructions. Protein levels were normalized to total protein concentration. The assay was run as 3 replicates in total.

### 4.5 Cell counting kit—8 (CCK8) assay

Cell proliferation was analyzed using CCK8 assay (Sigma-Aldrich, catalog no. 96992) to quantitate the number of viable cells. In the first experiment, 500 cells from each group (A549, vector, CXCL17) were seeded into 96-well plates The assay was repeated at two independent experiments with a total of 5 replicates. 10 μL of CCK8 solution was added into each well 24 and 48 h after seeding and incubated for 4 h at 37°C. The absorbance value was read at 450 nm using a microplate reader (Thermo Scientific Varioskan Flash).

### 4.6 Wound healing assay

In 6-well plates, 350.000 cells from each group (A549, vector, CXCL17) were plated in high glucose DMEM containing 2% FBS as a total of as a total of 3 replicates in first trial, while in the second trial, 2 replicates were performed. The experiment was completed with a total of 5 replicates in two separate times. A 200 μL pipette tip was used to scratch the cell monolayer’s surface in the center of the wells after 24 h had passed and it had reached 80% confluence. 24 and 48 h after creating the scratch, 10 photographs were taken with an EVOS M500 microscope on 10 × and the wound area was calculated using ImageJ wound healing size tool at every time point.

### 4.7 Matrigel invasion assay

24-well Transwell plates with 0.8um membrane inserts (Corning^®^ Costar^®^ Transwell^®^ cell culture inserts, catalog no. CLS3464) were coated with 50 μL of 1:50 diluted Matrigel (Corning^®^ Matrigel^®^ Basement Membrane Matrix, LDEV-free, 10 mL, catalog no. 354234) with high glucose DMEM and then incubated at 37°C for 1 h to gel. The surface was aspirated to remove the non-gelling solution. 10.000 cells from each group (A549, vector, CXCL17) were seeded into the upper chamber in high glucose DMEM containing 1% FBS in 2 replicates at 2 independent experiments with 4 total replicates. High glucose DMEM containing 10% FBS medium was added to the lower chamber to attract cells. One of these plates was set up to be incubated for 24 h, and the other for 48. After 24 and 48 h, cells invade to the bottom chamber were fixed using ice cold 100% methanol for 10 min and stained with Giemsa stain (Merck, catalog no. 1.09204.0500) for 5 min. Photographs of invaded cells were taken with an EVOS M500 microscope on 10X and analyzed with ImageJ.

### 4.8 Statistical analysis

All experiments were conducted at least twice, and the Graphpad Prism 9 program was used to perform all statistical analyses. The results were displayed as the mean ± standard deviation. One-way analysis of variance (ANOVA) was performed to assess the differences of mRNA and protein levels of each group using Tukey’s multiple comparisons test. Two-way ANOVA was performed to assess the differences of proliferated and invaded cells using Šídák’s multiple comparisons test. Two-way ANOVA was performed to assess the differences of migrated cells using Tukey’s multiple comparisons test. The threshold for significance was set at *p* < 0.05.

## Data Availability

The original contributions presented in the study are included in the article/Supplementary material, further inquiries can be directed to the corresponding author.
